# Unusual presentation of talonavicular joint pigmented villonodular synovitis: a case report

**DOI:** 10.1186/s13256-024-04385-7

**Published:** 2024-02-19

**Authors:** Omid Elahifar, Ali Torkaman, Moein Ghaeini, Arvin Eslami

**Affiliations:** 1 Department of Orthopedic, School of Medicine, Iran University of Medical Sciences, Tehran, Iran; 2 Bone and Joint Reconstruction Research Center, Department of Orthopedics, School of Medicine, Iran University of Medical Sciences, Tehran, Iran

**Keywords:** PVNS, Pigmented villonodular synovitis, Pigmented villonodular, Talonavicular, Synovitis, Case report

## Abstract

**Background:**

Pigmented villonodular synovitis is a rare yet locally invasive disorder impacting synovial tissues. This case report delineates the atypical manifestation of pigmented villonodular synovitis in the talonavicular joint, detailing its diagnostic complexity and successful management.

**Case presentation:**

A 56-year-old Iranian patient with a 4-year history of chronic ankle pain, initially diagnosed with degenerative joint disease post-trauma based on imaging, underwent talonavicular fusion surgery. An unexpected pigmented villonodular synovitis mass was encountered during the procedure. Subsequent interventions encompassed tumor resection, talonavicular joint fusion, and allograft bone grafting. Despite the initial intervention, persistent pain and nonunion necessitated a secondary procedure, involving joint surface curettage and autograft bone grafting. At the 12-month follow-up, the patient remained pain-free without tumor recurrence.

**Conclusion:**

This case report highlights the significance of considering pigmented villonodular synovitis as a crucial differential diagnosis in chronic ankle pain, even when there is evidence of degenerative joint disease and a history of trauma. Magnetic resonance imaging serves a crucial role in accurate diagnosis. Treatment necessitates precise tumor removal, appropriate bone grafting techniques and secure fixation.

Level of evidence: IV.

## Introduction

Pigmented villonodular synovitis (PVNS) is an uncommon, benign, yet locally aggressive disorder that primarily affects the synovium of joints, bursae, and tendon sheaths [1]. PVNS can be classified into two distinct types: diffuse and localized [1, 2]. The localized form is characterized by the presence of a pedunculated nodule within the joint, whereas the diffuse type is associated with infiltrative synovial involvement that leads to bone erosion [3]. The annual incidence of PVNS is reportedly at 1.8 cases per million people [4], with most patients typically affected during their third or fourth decade of life [5]. Involvement of the foot and ankle occurs in less than 10% of patients with PVNS [6–9]. Surgical excision is a commonly employed treatment for PVNS, although the specific surgical approach may vary based on the type and anatomical location of the condition [5].

To our knowledge, only one case of talonavicular PVNS with associated swelling and discomfort has been documented [10]. However, no case of PVNS management in the talonavicular joint through excision, fusion, and bone grafting has been reported, notably following an initial diagnosis of talonavicular degenerative joint disease (DJD) post-trauma.

This case report describes a patient initially diagnosed with talonavicular joint DJD, where a suspicious mass indicative of PVNS was discovered during surgery.

## Case presentation

A 56-year-old Iranian male farmer, who was a smoker and occasionally used opium orally, but had no history of alcohol consumption, visited our center reporting intermittent pain in his left ankle, primarily during physical activity. He disclosed a prior bicycle-related ankle injury 4-years earlier, which had not been formally assessed or treated. Post-injury, he began experiencing persistent ankle pain. The patient had no other significant past medical, social, environmental, or family history, and his employment involved routine agricultural work.

### Physical examination

Upon admission, a comprehensive physical examination was conducted. The patient’s vital signs were recorded, showing a pulse rate of 81 beats per minute, blood pressure of 110/85 mmHg, and an oral temperature of 37.10°C. The examination of the lower extremities revealed that the range of motion in the tibiotalar joint was within normal limits, and there was no evidence swelling or erythema. Palpation of the left hindfoot, particularly in the talonavicular region, elicited tenderness, suggesting localized pathology. Neurological assessment of the lower extremities did not reveal any sensory deficits, and motor function was intact. Reflexes were normal and symmetrical in both lower limbs. No signs of systemic involvement, such as fever or lymphadenopathy, were noted.

### Laboratory findings on admission were as follows

- Complete blood count (CBC)
  o White blood cell (WBC) count: 5600 cells/µL
  o Hemoglobin (Hb): 13.7 g/dL
  o Platelet (PLT) count: 204,000 cells/µL
- Coagulation profile:
  o Prothrombin time (PT): 14 seconds
  o International normalized ratio (INR): 1.04
- Inflammatory markers:
  o Erythrocyte sedimentation rate (ESR): 41 mm/hour
  o C-reactive protein (CRP): 2 mg/L
- Urinalysis: results were within normal limits.
- Liver function tests (LFTs): all parameters were within normal ranges.

### Radiological findings

**Fig. 1:**
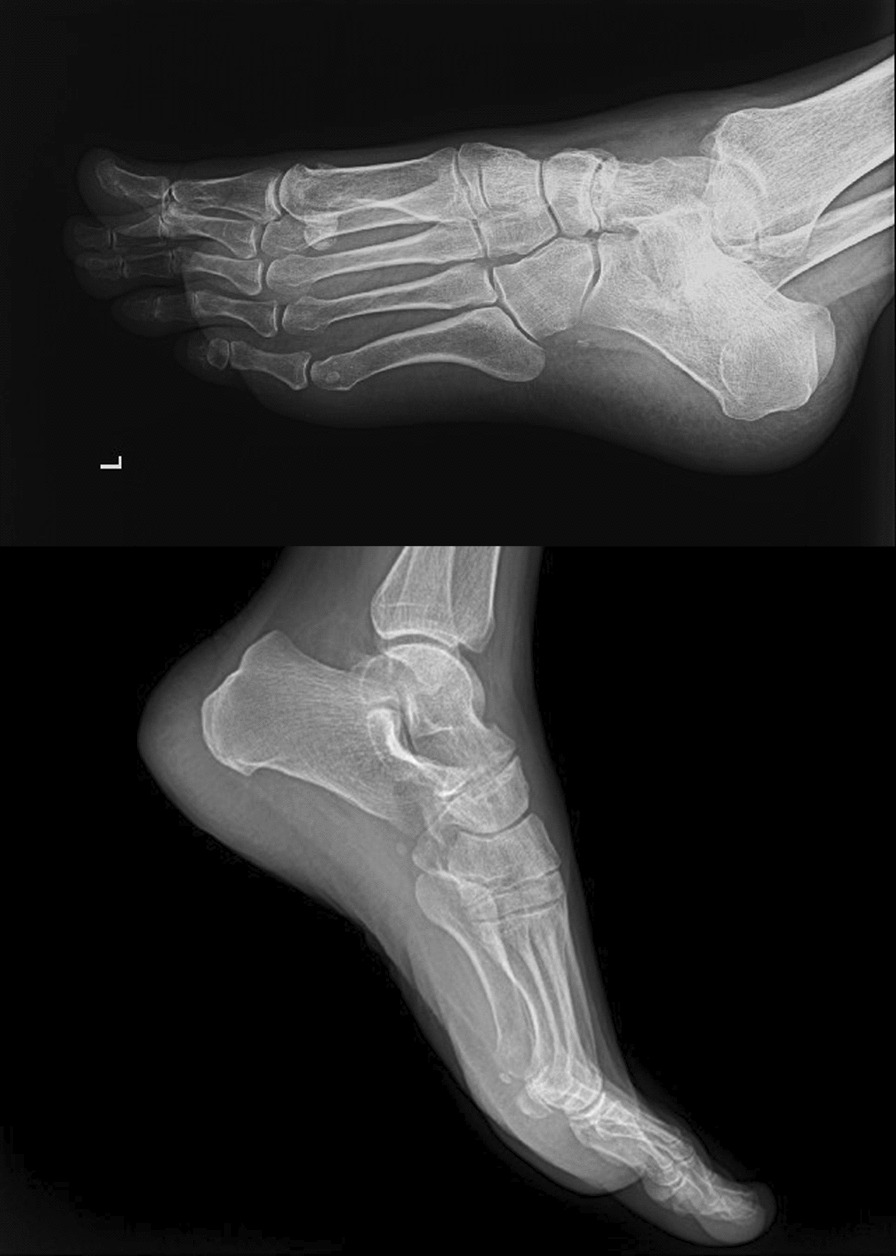
Oblique and lateral radiography showing cortical erosion and sclerosis and joint space narrowing in the talonavicular joint

**Fig. 2:**
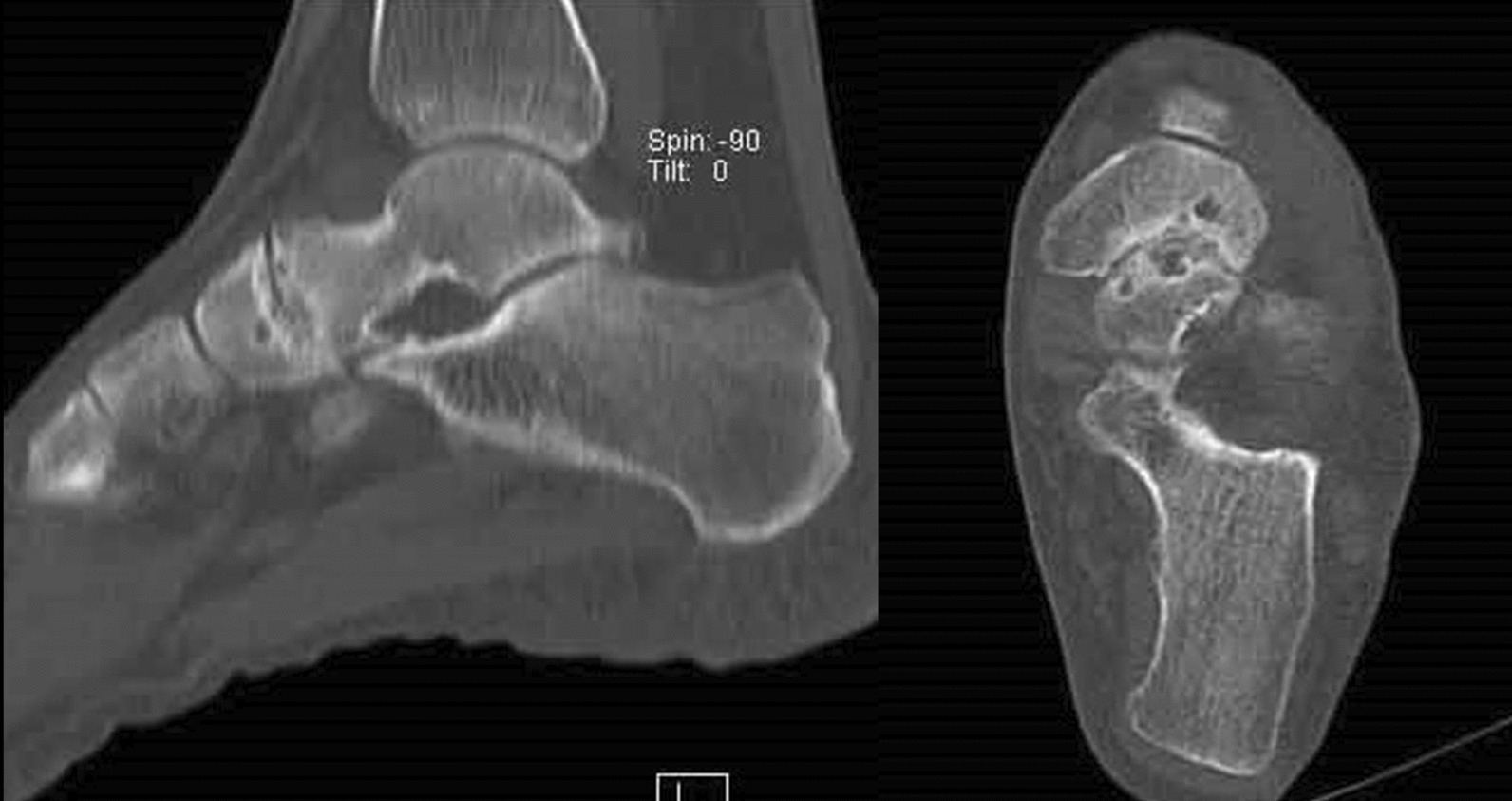
Sagittal and coronal CT scans showing cortical erosion and sclerosis, joint space narrowing and subchondral bone cysts in the talonavicular joint

X-ray imaging of the left foot and ankle revealed diffuse lytic areas within the subchondral bone on both sides of the talonavicular joint (Fig. 1). A computed tomography (CT) scan revealed a substantial reduction in the left talonavicular joint space, along with cystic changes, severe sclerosis around the joint, and degenerative alterations in the subtalar joint (Fig. 2).

### First surgical intervention

**Fig. 3:**
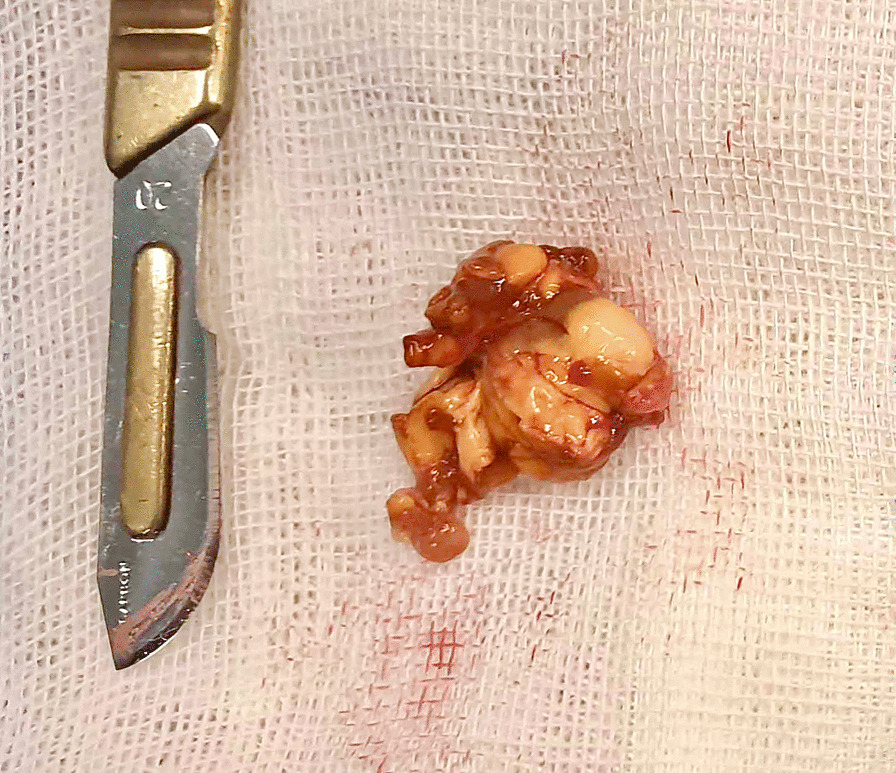
The gross pathology of the excised lesion revealed a dark brown, multinodular mass with areas of yellow discoloration

Given the initial diagnosis of DJD subsequent to the history of trauma, fusion was considered a viable treatment option. An anterior approach was employed to access the talonavicular joint. Upon exploration of the joint, a pedunculated brown encapsulated and lobulated mass measuring 3 cm × 2 cm within the interarticular space was identified, raising a strong suspicion of PVNS. A local excisional biopsy of the mass was performed. Subsequently, due to subchondral erosion, joint space narrowing, and persistent pain, fusion of the talonavicular joint was performed using a miniplate, supplemented by the application of cancellous allografts. The excised tissue was sent for pathological examination due to concerns about potential PVNS involvement (Fig. 3).

### Pathological examination

**Fig. 4:**
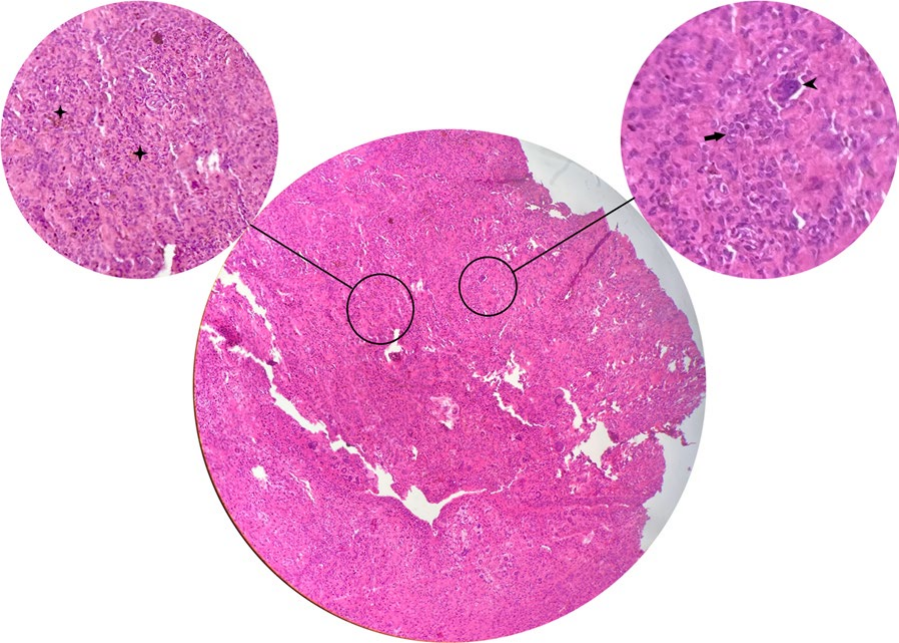
Histology was performed with hematoxylin–eosin (H–E) staining, and the brown areas indicate extensive hemosiderin deposition (stars). Multinucleated giant cells (arrowheads) and mononuclear histiocytoid cells (straight arrow) are visible

The lesion presented as a well-defined nodular mass comprising numerous giant cells and stromal cells. Additionally, there were segments of synovial tissue with villous projections, and extensive hemosiderin accumulation was observed in association with foamy macrophages. The histological diagnosis confirmed the presence of PVNS in the talonavicular joint (Fig. 4). Additionally, microbiological cultures conducted on the tumor specimen returned negative.

### Hospital course and treatment in first hospital admission

During his initial hospital stay following the surgical procedure, the patient received the following treatments:

- Cefazolin: administered intravenously, 1 g every 8 hours, for the duration of 1 day postoperatively.
- Low molecular weight heparin (enoxaparin): administered subcutaneously at a dose of 40 mg once daily, starting postoperation and continued for the 2-day duration of the hospital stay.

### Follow-up after the first surgery

Postoperatively, the patient was prescribed aspirin (81 mg orally, twice daily) for 6 weeks and acetaminophen (500 mg orally, as needed, up to twice daily) for pain relief. Follow-up visits were initially scheduled 2 weeks after discharge, followed by monthly assessments.

A total of 10 months after the initial surgery, the patient experienced persistent pain in the same location. Serial X-rays indicated nonunion, which led to the decision for a second surgical intervention.

### Second surgical intervention

For the second surgical intervention, an incision was made at the site of the prior scar, situated anterior to the ankle. Upon accessing the talonavicular joint, oligotrophic nonunion was identified, and no evidence of tumor recurrence was observed. The joint surface was meticulously reconditioned using a curette, and the defect was comprehensively filled with cancellous autografts harvested from the iliac crest. The miniplate was subsequently repositioned.

### Hospital course and treatment in second hospital admission

During the second hospitalization, the patient received:

- Cephazolin: administered intravenously, 1 g every 8 hours for 2 days postoperation.
- Low molecular weight heparin (enoxaparin): 40 mg subcutaneously once daily during the 3-day hospital stay.

### Follow-up of the second surgery

**Fig. 5:**
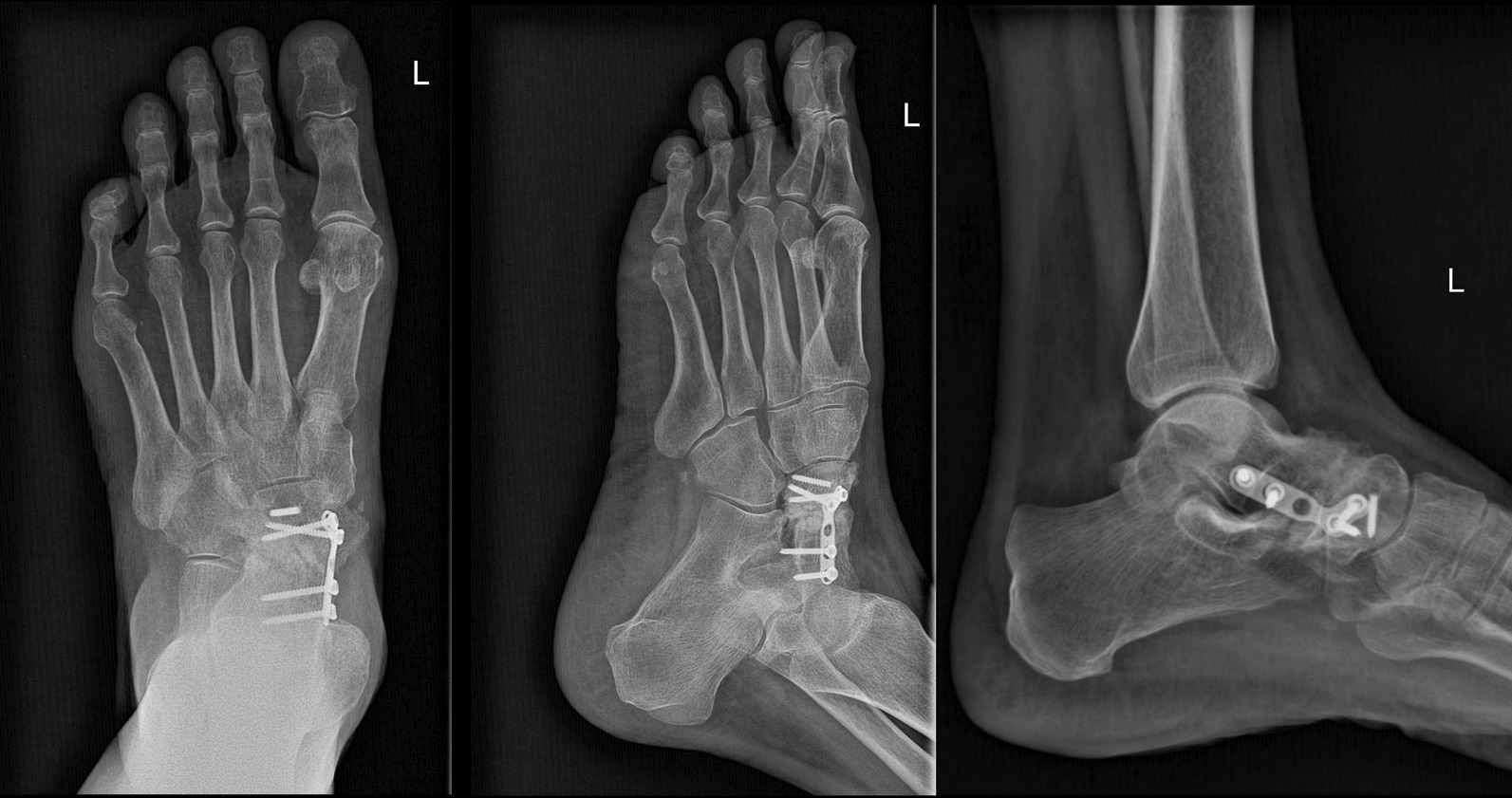
Anterioposterior (AP), lateral, oblique radiography showing union in the talonavicular joint 12 months after the second surgery

Following the second surgical procedure, the patient was prescribed aspirin (81 mg orally, twice daily for 6 weeks) and acetaminophen (500 mg orally as needed, up to twice daily) for pain relief. During subsequent follow-up visits, which were scheduled initially 2 weeks after discharge and then monthly, acetaminophen remained the only prescribed medication, taken as required for pain management. A total of 12 months after the second surgical procedure, the patient remained pain-free, with conclusive evidence of complete union in the talonavicular joint (Fig. 5). The results of the Foot and Ankle Outcome Score (FAOS) questionnaire [11] were as follows: FAOS symptoms: 96, FAOS pain: 100, FAOS activities of daily living (ADL): 100, FAOS sports/recreation: 95, and FAOS quality of life (QOL): 100.

## Discussion

In this case report, we describe the successful management of a middle-aged patient who presented with PVNS involving the talonavicular joint. The treatment strategy involved tumor excision, fusion of the talonavicular joint, and autograft bone grafting.

Although PVNS in the foot and ankle is rare, it can often mimic the features of DJD, particularly in patients exhibiting subchondral erosions, posing a diagnostic challenge [9, 12–14]. It is crucial to maintain a high level of suspicion of PVNS in middle-aged patients displaying DJD symptoms in the foot and ankle joints, even when there is a history of trauma [14].

The clinical presentation of PVNS is often nonspecific, typically manifesting as a painless, firm, and immobile soft tissue mass [13, 14]. However, diagnosing PVNS can be challenging, as demonstrated by our patient who experienced ankle pain despite a history of trauma. This case emphasizes the pivotal role of imaging in diagnosing PVNS, especially when clinical symptoms are nonspecific.

In chronic cases, X-ray and CT scans may reveal erosion of the sclerotic cortex near the ankle joint, alongside the soft tissue mass shadow. However, these findings lack specificity, as observed in our patient, where there was no evidence of a soft tissue mass in the physical examination or imaging before surgery [15]. Magnetic resonance imaging (MRI) is considered the modality of choice for PVNS diagnosis [12]. On MRI, PVNS typically presents as a combination of high signal intensity with localized areas of low signal intensity in T2-weighted images and low to moderate signal intensity in T1-weighted images. [6, 15, 16]

In our patient, the initial clinical presentation, which included degenerative features and a history of trauma, initially prompted an investigation into posttraumatic DJD and led to the assumption that an MRI was unnecessary. This highlights the importance of considering MRI as an essential diagnostic step before surgery in similar cases.

Surgical excision is a frequently employed therapeutic approach for treating PVNS; however, the specific treatment may vary according to the type and anatomical location of the condition. [5] For localized PVNS, most studies advocate complete resection of the lesion, and curettage of bone cysts or sites of bone erosion, which was the chosen surgical approach in our case [13, 14, 17]. In patients with diffuse PVNS, incomplete resection, or local recurrence, adjunctive and neoadjuvant treatments such as radiation therapy and cryotherapy are recommended [18–20]. However, in our patient, the persistence of symptoms after surgery was attributed to nonunion rather than tumor recurrence.

Recurrence is a significant concern in PVNS, with reported rates reaching up to 50%. It commonly occurs when synovial tissue excision is incomplete or in cases of the diffuse type, which inherently poses a higher risk of recurrence [19]. During the 12-month follow-up after the second surgery, no clinical evidence of lesion recurrence was observed. However, given the possibility of recurrence at longer intervals, regular clinical examinations and imaging evaluations at 6-month intervals are recommended. [14]

### Strengths

1. Unique presentation: this case report sheds light on an unusual presentation of PVNS in the talonavicular joint, offering insights into a rare manifestation.
2. Comprehensive management: the report details a comprehensive treatment approach involving tumor resection, joint fusion, and bone grafting, providing valuable clinical implications.

### Limitations

1. Single case: this study’s scope is restricted by its reliance on a single patient, limiting the applicability of the findings to a broader population.
2. Use of MRI: MRI was not utilized in the early stages of the disease due to the possibility of misdiagnosis with DJD. Additionally, MRI was not employed before the second surgery due to concerns about potential metal artifacts from the implant.

## Conclusion

In middle-aged patients with persistent ankle pain and radiographic evidence suggestive of DJD, considering the possibility of tumors, notably PVNS, is crucial. MRI aids in accurate differential diagnosis. The optimal approach involves precise tumor removal and appropriate bone grafting techniques, particularly autografts, along with secure fixation. While this comprehensive treatment strategy appears promising for effective patient care, further research is necessary to refine the management of such cases.

## Data Availability

The data are available and can be provided upon reasonable request. Interested researchers may contact the corresponding author to gain access to the data.

## Abbreviations

ADL: Activities of daily living
CT: Computed tomography
DJD: Degenerative joint disease
FAOS: Foot and ankle outcome score
MRI: Magnetic resonance imaging
QOL: Quality of life

## References

[1] Granowitz SP, Mankin HJ (1967). Localized pigmented villonodular synovitis of the knee. Report of five cases. J Bone Joint Surg Am.

[2] Sciot R, Rosai J, Dal Cin P (1999). Analysis of 35 cases of localized and diffuse tenosynovial giant cell tumor: a report from the Chromosomes and Morphology (CHAMP) study group. Mod Pathol.

[3] Chou LB, Ho YY, Malawer MM (2009). Tumors of the foot and ankle: experience with 153 cases. Foot Ankle Int.

[4] Myers BW, Masi AT (1980). Pigmented villonodular synovitis and tenosynovitis: a clinical epidemiologic study of 166 cases and literature review. Medicine (Baltimore).

[5] Flandry F, Hughston JC (1987). Pigmented villonodular synovitis. J Bone Joint Surg Am.

[6] Friscia DA (1994). Pigmented villonodular synovitis of the ankle: a case report and review of the literature. Foot Ankle Int.

[7] Ghert MA, Scully SP, Harrelson JM (1999). Pigmented villonodular synovitis of the foot and ankle: a review of six cases. Foot Ankle Int.

[8] Kaneko K, Nakahara D, Tobe M (2000). Pigmented villonodular synovitis of the ankle in an adolescent. Int Orthop.

[9] Kubat O, Bojanić I, Smoljanović T (2017). Localized pigmented villonodular synovitis of the ankle: Expect the unexpected. Foot Ankle Surg.

[10] Okoro T, Isaac S, Ashford RU, Kershaw CJ (2009). Pigmented villonodular synovitis of the talonavicular joint: a case report and review of the literature. Foot (Edinb).

[11] Roos EM, Brandsson S, Karlsson J (2001). Validation of the foot and ankle outcome score for ankle ligament reconstruction. Foot Ankle Int.

[12] Duncan N, Rajan R (2015). Case report of pigmented villonodular synovitis arising from the calcaneocuboid joint in a 12 year old male. Foot (Edinb).

[13] Carpintero P, Gascon E, Mesa M, Jimenez C, Lopez U (2007). Clinical and radiologic features of pigmented villonodular synovitis of the foot: report of eight cases. J Am Podiatr Med Assoc.

[14] Ebrahimpour A, Sadighi M, Jafari Kafiabadi M, Chehrassan M, Biglari F (2021). Pigmented villonodular synovitis arising from calcaneocuboid joint in an army staff: a case report. Arch Bone Jt Surg.

[15] Freedman BA, Lin DL, Tis JE (2007). Pigmented villonodular synovitis of the calcaneocuboid joint in an 11-year-old child with subtalar coalition. Foot Ankle Int.

[16] Friedman T, Chen T, Chang A (2013). MRI diagnosis of recurrent pigmented villonodular synovitis following total joint arthroplasty. HSS J.

[17] Novikov D, Richardson MW, Ho C, Gould ES, Khan FA (2018). A rare incidence of pigmented villonodular synovitis of the ankle in an adolescent. J Foot Ankle Surg.

[18] Muramatsu K, Iwanaga R, Tominaga Y, Hashimoto T, Taguchi T (2018). Diffuse pigmented villonodular synovitis around the ankle. J Am Podiatr Med Assoc.

[19] Bisbinas I, De Silva U, Grimer RJ (2004). Pigmented villonodular synovitis of the foot and ankle: a 12-year experience from a tertiary orthopedic oncology unit. J Foot Ankle Surg.

[20] Segler CP (2003). Irradiation as adjunctive treatment of diffuse pigmented villonodular synovitis of the foot and ankle prior to tumor surgical excision. Med Hypotheses.

